# *GUCY2D*-Related Retinal Dystrophy with Autosomal Dominant Inheritance—A Multicenter Case Series and Review of Reported Data

**DOI:** 10.3390/genes13020313

**Published:** 2022-02-08

**Authors:** Jonas Neubauer, Leo Hahn, Johannes Birtel, Camiel J. F. Boon, Peter Charbel Issa, M. Dominik Fischer

**Affiliations:** 1 Centre for Ophthalmology, University Hospital Tuebingen, University of Tuebingen, 72076 Tuebingen, Germany; presse@med.uni-tuebingen.de; 2 Department of Ophthalmology, Amsterdam UMC, Academic Medical Center, 1105 AZ Amsterdam, The Netherlands; l.hahn@amsterdamumc.nl (L.H.); camiel.boon@amsterdamumc.nl (C.J.F.B.); 3 Oxford Eye Hospital, Oxford University Hospitals NHS Foundation Trust, Oxford OX3 9DU, UK; Johannes.Birtel@ouh.nhs.uk (J.B.); study-enquiry@outlook.com (P.C.I.); 4 Nuffield Laboratory of Ophthalmology, Department of Clinical Neurosciences, University of Oxford, Oxford OX3 9DU, UK; 5 Department of Ophthalmology, University Hospital Bonn, University of Bonn, 53127 Bonn, Germany; 6 Department of Ophthalmology, Leiden University Medical Center, 2333 ZA Leiden, The Netherlands

**Keywords:** GUCY2D, guanylate cyclase 2D, inherited retinal disease, cone dystrophy, cone–rod dystrophy, visual acuity, disease progression, eye, OCT, symmetry

## Abstract

To report the clinical phenotype and associated genotype of a European patient cohort with *GUCY2D*-related autosomal-dominant (AD) cone–/cone–rod dystrophy (COD/CORD), we retrospectively analyzed 25 patients (17 female, range 12–68) with *GUCY2D*-related AD-COD/CORD from three major academic centers in Europe and reviewed the previously published data of 148 patients (visual acuity (VA), foveal thickness, age of first symptoms, and genetic variant). Considering all the patients, the onset of first symptoms was reported at a median age of 7 years (interquartile range 5–19 years, *n* = 78), and mainly consisted of reduced VA, photophobia and color vision abnormality. The disease showed a high degree of inter-eye symmetry in terms of VA (*n* = 165, Spearman’s ρ = 0.85, *p* < 0.0001) and foveal thickness (Spearman’s ρ = 0.96, *n* = 38, *p* < 0.0001). Disease progression was assessed by plotting VA as a function of age (*n* = 170). A linear best-fit analysis suggested a loss of 0.17 logMAR per decade (*p* < 0.0001). We analyzed the largest cohort described so far (*n* = 173), and found that the most common mutations were p.(Arg838Cys) and p.(Arg838His). Furthermore, the majority of patients suffered severe vision loss in adulthood, highlighting a window of opportunity for potential intervention. The emerging patterns revealed by this study may aid in designing prospective natural history studies to further define endpoints for future interventional trials.

## 1. Introduction

Inherited retinal diseases (IRD) are a group of heterogeneous disorders caused by mutations in over 250 different genes that are important for retinal function [1]. A subgroup of these are autosomal-dominant (AD) cone–/cone–rod dystrophies (COD/CORD), which primarily affect cones, while rods may degenerate later and to a varying extent [2]. Therefore, the initial symptoms in COD/CORD typically include a decline in visual acuity (VA), photophobia, dyschromatopsia, and reading difficulties due to a central scotoma [2,3]. Only subtle RPE changes may be observed in the early phase of the disease, which can later progress to bull’s eye maculopathy and, finally, to central atrophy [4,5]. Electrophysiological examination often describes a shift in implicit time at the 30 Hz flicker responses in the initial phase, and a decrease in the amplitudes of both a- and b-waves in photopic responses during the course of the disease [2].

Mutations in *GUCY2D* are among the most common causes of AD COD/CORD [6,7,8]. Its gene product is retinal guanylate cyclase 2D (RetGC-1), which is crucial in the phototransduction process of cones and rods. After stimulation by photons, changes in ion flows lead to hyperpolarization of the photoreceptor cells. The RetGC-1 protein plays a pivotal role in restoring the original depolarized (dark-adapted) state of photoreceptors through the production of cGMP, thereby indirectly opening cGMP-dependent ion channels, which, in turn, leads to depolarization of the photoreceptors.

There is substantial phenotypic heterogeneity of *GUCY2D*-related retinal disease. Severe loss-of-function variants on both alleles result in autosomal recessive Leber congenital amaurosis type 1 (LCA1) [9,10]. In contrast, AD COD/CORD is usually associated with mutations in codon 838, or those in close proximity to it. The resulting protein seems to be functional, but has altered Ca^2+^ sensitivity of RetGC-1, leading to a later onset of symptoms, usually in childhood or adolescence [10].

The aim of this study was to report the data of patients with *GUCY2D*-related AD COD/CORD from three major academic tertiary IRD referral centers in Europe, and to compare them with information that has already been published.

## 2. Methods

Out of the identified 25 patients with *GUCY2D*-related AD COD/CORD, nine patients (2, 3, 13, 14, 15, 16, 22, 23 and 24) underwent a clinical examination at the Ophthalmology Department of the University Hospital of Tübingen, six patients were examined at the Department of Ophthalmology at Amsterdam University Medical Centers (patients 4, 17, 18, 19, 20, 21), and 10 patients were examined at the Department of Ophthalmology, University Hospital Bonn (patients 1, 5, 6, 7, 8, 9, 10, 11, 12, 25). Six patients were examined more than once. Of these, only one set of results, from the day with the most complete examinations, was used for further analysis. Our chart review collected records of visual acuity, intraocular pressure, optical coherence tomography and fundus autofluorescence imaging (both, Spectralis HRA + OCT, Heidelberg Engineering, Germany), fundus photography, visual field (Octopus 900, Haag-Streit, Germany), and a Farnsworth panel D-15 color examination. Clinical information of patients 2, 3, 13, 14, 15, 22, 23, and 24 has partly been described in previous reports [5,11]. Two members of family five (patients 17 and 21) were part of a publication in 1992, before the causative gene and variant were known [12].

Low visual acuity measurements such as counting fingers and hand movement were converted to logMAR 1.98 and logMAR 2.28, respectively (Lange et al., 2009). Two patients with light perception in their left eye only were excluded from the analysis of visual acuity symmetry.

The study was conducted according to the guidelines of the Declaration of Helsinki, and was approved by the Ethics Committee of the University of Tübingen, Germany (149/2018BO2, 21 March 2018).

For the review of previously published clinical data on *GUCY2D*, a MEDLINE/PubMed search was performed on 1 May 2020 (gucy2d eye, gucy2d dystrophy) and all publications with available clinical data have been included for further analysis (20 out of 112 publications) [11,13,14,15,16,17,18,19,20,21,22,23,24,25,26,27,28,29,30,31]. For statistical analysis and creation of graphs, Graph Pad Prism 6 as well as R version 3.6.0 were used.

## 3. Results

### 3.1. Patient Cohort Characteristics

**Figure 1:**
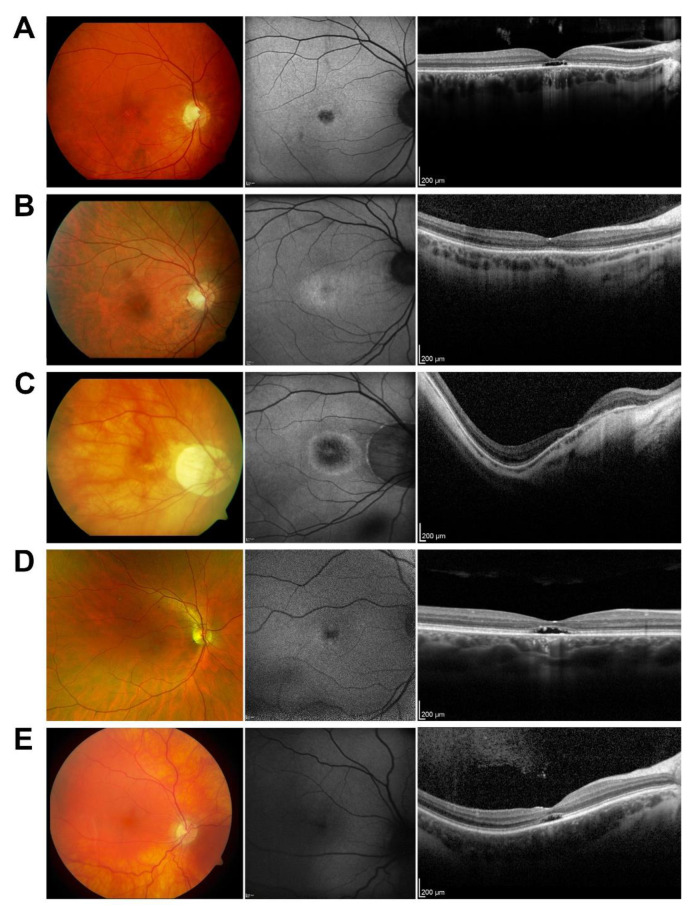
Fundus color picture, fundus autofluorescence and OCT scan of study patients. (**A**) ID 13 p.(Arg838Cys) (45-year-old female, logMAR 1.3, central reduced autofluorescence; first symptoms: vision loss at the age of 19). (**B**) ID 14 p.(Arg838Cys) (34-year-old male, logMAR 0.3, bull’s eye configuration in autofluorescence; first symptoms: vision loss). (**C**) ID 15 p.(Arg838Cys) (58-year-old female, logMAR 1.1, central reduced autofluorescence; first symptoms: color vision distortion and photophobia at the age of 54). (**D**) ID 16 p.(Arg838Cys) (41-year-old male, logMAR 1.1, central reduced autofluorescence; first symptoms: vision loss from birth on). (**E**) ID 24 p.(Arg838His) (26-year-old female, logMAR 1.3, normal autofluorescence).

In this study, 25 patients (17 female) with AD COD/CORD associated with mutations in *GUCY2D* were included. The median age upon examination was 36 years (range 12–68). Fourteen patients from five families were related; no consanguinity was known. The ages at the onset of the first symptoms ranged from the first weeks of life to 54 years of age (data available for 21 patients). The symptoms at subjective disease onset included deterioration of visual acuity (17/21), glare/photophobia (3/21), and color vision abnormalities (2/21). Visual acuity at presentation ranged from logMAR 0.00 to hand movement. Six patients were examined more than once and their visual acuity values over time are presented in Supplementary Figure S3. Fundus autofluorescence imaging (*n* = 20) was abnormal in 19 out of 20 patients with central hyper- and hypo-fluorescence (9/20), central reduced autofluorescence (6/20), central increased autofluorescence (1/20), bull’s eye pattern (2/20), and an unspecified pattern was present in one patient (Supplementary Table S1 and Figure S2). The representative patients are shown in Figure 1.

### 3.2. Molecular Assessment

**Figure 2:**
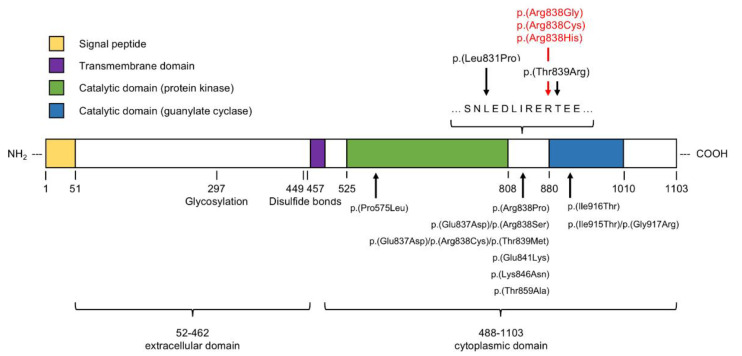
Schematic of the protein product as transcribed and translated from the *GUCY2D* gene. The extracellular domain follows a short signal peptide at the N-terminus and is subject to some posttranslational modifications (glycosylation and disulfide bonds). A short transmembrane domain links it to the cytoplasmic domain with enzymatically active domains (green and blue). All disease-causing sequence variants are located between those domains, between amino acids 830 and 840 (arrows and denominations top right). On the bottom, additional known mutations causing AD COD/CORD are indicated [13,14,15,16,17,18,19,20,21,22,23,24,25,26,27,28,29,30,31,32,33].

Genetic analysis identified five different *GUCY2D* variants in the 25 patients included. Thirteen patients (52%) were revealed to have the c.2512C>T mutation (p.(Arg838Cys)), and eight patients (32%) had the c.2513G>A mutation (p.(Arg838His)); two of those patients had an additional sequence variant of unknown significance in *GUCA1B,* which was also observed in some healthy family members. Two patients were shown to have the variant c.2512C>G (p.(Arg838Gly)), and the c.2492T>C (p.(Leu831Pro)) variant was identified in one patient. One patient showed two sequence variants in the *GUCY2D* gene (c.74C>T, c.2516C>G; p.(Ser25Phe) and p.(Thr839Arg)). The point mutation at the N-terminal end of the protein product only affects the signal peptide of *GUCY2D* (Figure 2).

### 3.3. Disease Symmetry between Eyes

**Figure 3:**
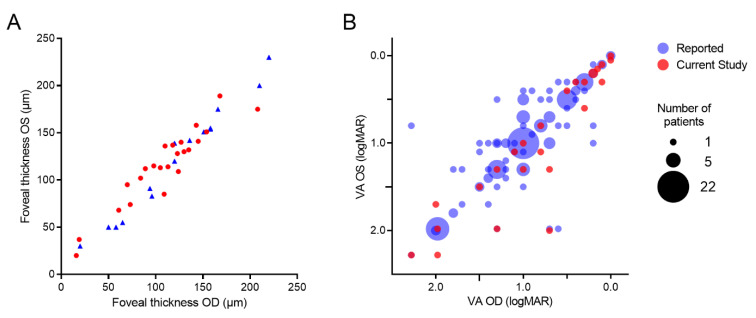
Analysis of symmetry between OD and OS. (**A**) High symmetry of foveal thickness in *n* = 38 patients (Spearman’s rho = 0.96). (**B**) High symmetry of visual acuity in *n* = 165 patients (Spearman’s rho = 0.85).

Both the structural and functional characteristics showed high symmetry (Figure 3). The analysis of anatomical endpoints between both eyes showed high symmetry for the retinal volume of the central 1 mm (available in 22 patients) (Spearman’s ρ = 0.96, *n* = 22, *p* < 0.0001), and for foveal thickness (available in 23 patients of our cohort and 15 published patients [15]) (Spearman’s ρ = 0.96, *n* = 38, *p* < 0.0001). The analysis of visual acuity was performed on 25 patients in this study, supplemented by the previously reported visual acuity data of 140 patients with *GUCY2D*-associated AD COD/CORD [11,13,14,15,16,17,18,19,20,21,22,23,24,25,26,27,28,29,30,31]. The median age of all the patients with VA data was 34 years (interquartile range 23–53 years). Visual acuity ranged from light perception to logMAR 0.00 (OD: median 1.0, interquartile range 0.5–1.3; OS: median 1.0, interquartile range 0.5–1.3) and showed a high level of symmetry (Spearman’s ρ = 0.85, *p* < 0.0001).

### 3.4. Disease Progression

**Figure 4:**
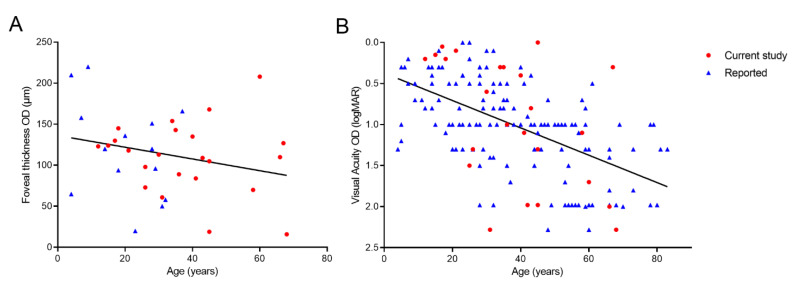
(**A**) Linear regression of foveal thickness (OD) associated with age (*n* = 38). (**B**) Linear regression of visual acuity values (OD) associated with age in *GUCY2D*-related AD COD/CORD patients (*n* = 170).

The disease progression of the patients in our cohort and the previously published *GUCY2D*-associated AD COD/CORD patients was assessed by plotting central foveal thickness (*n* = 38) and visual acuity (*n* = 170) as a function of age (Figure 4). A linear best fit analysis suggested a loss of 0.17 logMAR per decade (*p* < 0.0001). Furthermore, it is estimated that severe vision loss (logMAR > 1.0) is reached, on average, at the age of 38. More than 73% of the patients younger than 38 years of age had a better VA than 1.0 logMAR. Applying linear regression, the foveal thickness decreased by 7.2 µm per decade (*p* = 0.127). A similar decrease in VA and foveal thickness over time was found for left eyes (loss of 0.16 logMAR per decade (*p* < 0.0001)), where foveal thickness decreased by 5.1µm per decade (*p* = 0.261).

Comparing the two most common mutations (p.(Arg838Cys) and p.(Arg838His)), neither a significant difference in visual acuity (*p* = 0.99) nor in the disease progression rate (*p* = 0.08) were detected (Supplementary Figure S4).

**Figure 5:**
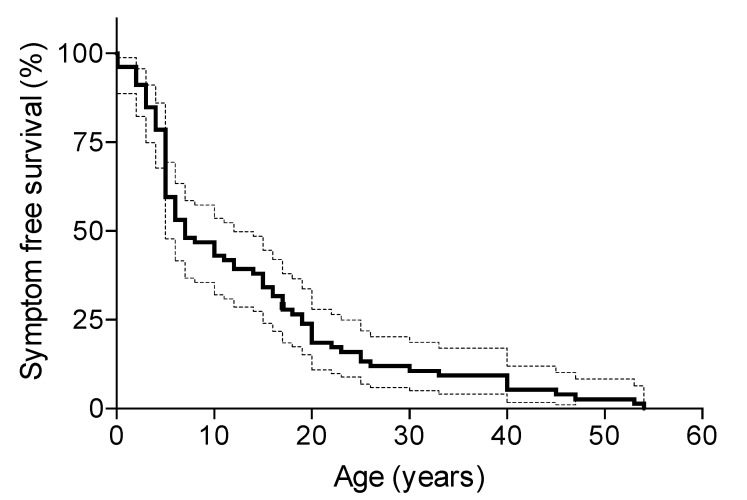
Kaplan–Meier survival curve for symptom-free survival (95% confidence interval) in *n* = 78 patients. Predicted incidence of first symptoms at a median age of 7.

The median time for symptom-free survival for all the patients (data available for *n* = 78 patients) was seven years (interquartile range 5–19 years) (Figure 5).

## 4. Discussion

We report the aggregated data of 173 patients with *GUCY2D*-related COD/CORD, a leading cause of AD COD/CORD. Considering the current efforts to develop gene-editing approaches, such information may be valuable for upcoming clinical studies [2,6,7,21,34].

The codon 838 of *GUCY2D* is considered a mutational hotspot due to its nucleotide sequence—CGC—which is prone to spontaneous deamination of methylated cytosine [35]. In codon 838, this would lead to p.(Arg838Cys) or p.(Arg838His) [21,35]. All but two of our 25 patients showed a mutation in this codon, with thirteen cases of p.(Arg838Cys), eight with p.(Arg838His), and two with p.(Arg838Gly) mutations. The genetic findings in our cohort are in line with previous reports (Supplementary Figure S1) [8,11,13,14,15,16,17,18,19,20,21,22,23,24,25,26,27,28,29,30,31,36].

Previous reports suggest that patients with p.(Arg838Cys) and p.(Arg838His) mutations typically present with a pure COD phenotype (i.e., without rod involvement), disease onset in late childhood or adolescence, increased light sensitivity (glare), and color vision abnormalities, but intact peripheral vision [21,31]. It is, therefore, of interest to consider the proposed disease mechanism. Previous studies reported that the RetGC-1 mutants p.(Arg838Cys) and p.(Arg838His) have an altered affinity for guanylyl cyclase-activating protein 1 and Ca^2+^, which, together, increases the activation of RetGC-1 at higher Ca^2+^ levels compared to the wild type [37,38]. Consequently, this may lead to an elevated Ca^2+^ concentration and death of photoreceptors via caspase activation and apoptosis [37,39]. Crucially, RetGC-1 is more strongly expressed in cone than in rod photoreceptors [40]. If cones were predominantly affected by the underlying disease mechanism, a phenotype primarily affecting cone photoreceptor function would be plausible. In our cohort, almost all the patients with p.(Arg838Cys) or p.(Arg838His) mutations showed color vision disorders, and the first reported symptom was a decline in visual acuity. However, only approximately half of these patients had normal peripheral visual fields (data available in 15 of 21 patients, eight of 15 showed normal peripheral visual fields) when they presented themselves, on average, 16 years (median 14.5 years) after the first symptoms (data available for 14 of 21 patients). Our findings support the notion that patients with p.(Arg838Cys) and p.(Arg838His) mutations share a similar phenotype and rate of disease progression.

In the patient with two variants of *GUCY2D* (c.74C>T, p.(Ser25Phe) and c.2516C>G, p.(Thr839Arg)), one is located immediately next to the 838 ‘hot spot’ codon (codon 839) on exon 13 and is deemed likely to be pathogenic by ClinVar (variation ID 635421). The other variant (p.(Ser25Phe)) is classified by ClinVar as likely to be benign (variation ID 444397). Our patient was a simplex case and their family members were not available for segregation analysis. Previous reports have interpreted the c.74C>T variant as not diseasecausing in two patients with a different composition of compound heterozygote sequence variants, as follows: c.74C>T (p.(Ser25Phe)) and c.2513G>A (p.(Arg838His)). It is currently unclear whether this c.74C>T (p.(Ser25Phe)) variant has an influence on the enzymatic activity and, consequently, on the photoreceptor function/survival, given the fact that the sequence variant is located in the N-terminal signal peptide domain [41]. In this context, we found it most appropriate to keep this patient in our analysis as a patient with autosomal dominant COD/CORD caused by the c.2516C>G, p.(Thr839Arg) variant.

So far, there is no treatment available for *GUCY2D*-associated COD/CORD. Even though progress has been made in the field of retinal gene therapy, most developments are based on the idea of introducing a normal coding sequence of the affected gene into the nucleus of the affected cells [42]. This, however, would most likely not be effective for *GUCY2D*-related COD/CORD because it will not address the presumed dominant-negative disease mechanism. However, gene-editing tools, such as CRISPR/Cas9, provide potential opportunities to disrupt the dominant allele. The first steps towards this goal have already been achieved by disrupting RetGC-1 expression in mice and macaque in vivo, using an adeno-associated virus delivered via CRISPR/Cas9 [34].

Appropriate controls are paramount for interventional clinical trials, which can be achieved by using inter-individual controls, or by using the fellow eye as an intra-individual control. Natural history studies show symmetry of the disease as the basis of a one-sided treatment, with the fellow eye serving as the internal control. This can be an attractive option in disease entities with large inter-individual variability due to, e.g., unknown environmental and/or additional genetic factors, which may be even more relevant for more multi-factorial diseases, such as age-related macular degeneration.

We have shown the inter-eye symmetry of VA and fovea thickness in patients with *GUCY2D*-associated AD COD/CORD. Since VA is a critical factor influencing the quality of life, and central retinal thickness is related to central visual function, we consider VA to be the most relevant endpoint. As cones are primarily affected in this disease and VA is a cone-driven visual function, this will also reflect disease progression and/or the efficacy of any treatment. The difficulty here lies in the linearity of photoreceptor degeneration. Once foveal cone photoreceptors have died, no significant VA gain is to be expected. There may only be a small window of opportunity to gain VA by treating dysfunctional cone photoreceptors before they die. Our analysis shows that VA decreases, on average, by about nine EDTRS letters per decade, leading to severe vision loss (logMAR > 1.0) by age 38.

The two main limitations of this study are the retrospective study design and the limited number of subjects. Despite the low prevalence of *GUCY2D*-related COD/CORD, we managed to pool data from 25 patients across multiple sites.

To our knowledge, in combination with the data from previous reports, this study provides an analysis of the largest combined cohort described so far, and emerging patterns can be used to design prospective natural history studies to help define endpoints for future interventional trials.

## 5. Conclusions

Supported by the data presented, it was shown that a sufficiently large therapeutic window exists for the majority of patients before severe visual impairment occurs. Moreover, future therapeutic treatments should focus on p.(Arg838Cys) and p.(Arg838His), as they are the most common variants of this rare disease. While our data cannot be used to infer the disease progression of individual patients, we were able to show that natural history studies and clinical interventions would require sufficiently long observation periods, and that the fellow eye could be used as an intra-individual control.

## Data Availability

The collected clinical data of the 25 patients is available in a Supplementary Table.

## Supplementary Materials

The following are available online at https://www.mdpi.com/article/10.3390/genes13020313/s1: Figure S1: overview of *GUCY2D* mutation frequency (*n* = 173). Figure S2: overview of different autofluorescence patterns. Figure S3: visual acuity values (OD and OS) associated with age in *GUCY2D*-related AD COD/CORD patients who have been examined more than once. Figure S4: linear regression of visual acuity values (OD) associated with age in patients with p.(Arg838Cys) (*n* = 85) and p.(Arg838His) (*n* = 38). Table S1: clinical data of 25 *GUCY2D*-related AD COD/CORD patients.
