# Supplementary material for: GUCY2D-Related Retinal Dystrophy with Autosomal Dominant Inheritance—A Multicenter Case Series and Review of Reported Data
Source: Genes (Basel). 2022 Feb 8;13(2):313. doi: 10.3390/genes13020313 (PMC8872159; doi:10.3390/genes13020313)
Supplement: Supplementary file 1 [file genes-13-00313-s001.zip › genes-1538777-supplementary.pdf]

Supplement

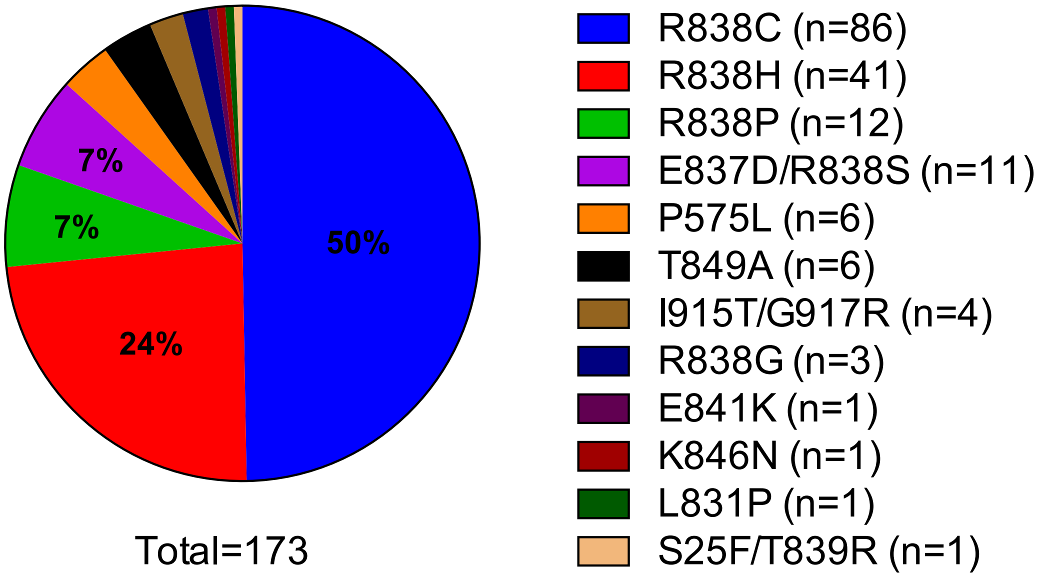

Supplemental Figure S1: Overview of *GUCY2D* mutation frequency (n = 173).

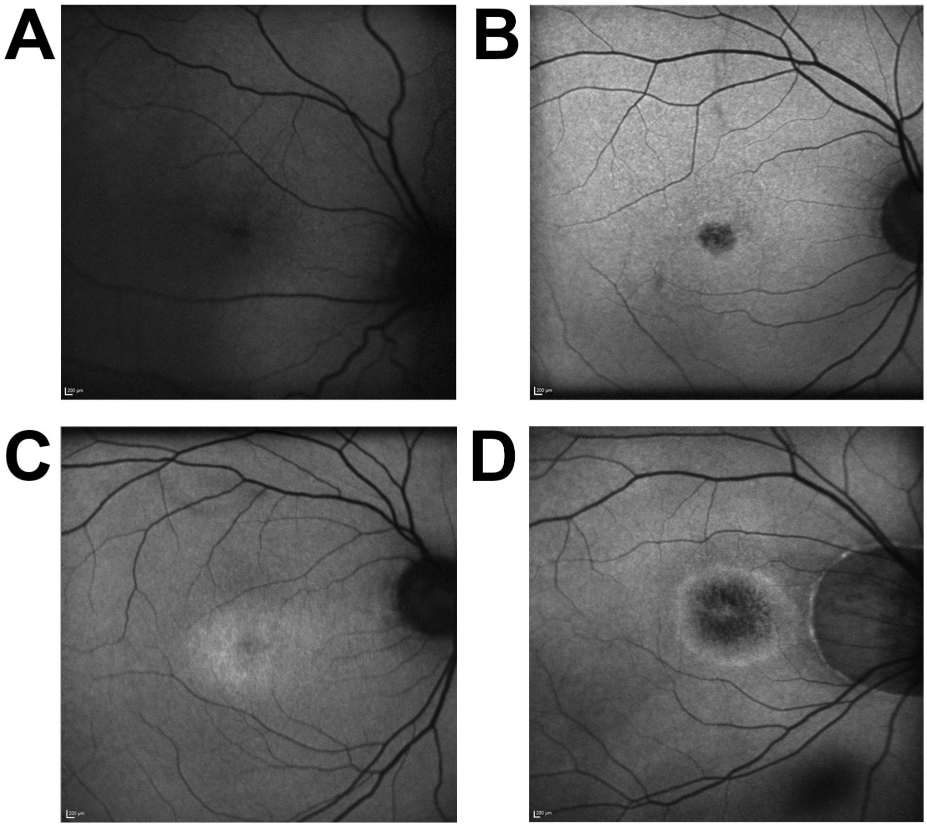

Supplemental Figure S2: Overview of different autofluorescence patterns. **A)** Normal autofluorescence. **B)** Central reduced autofluorescence. **C)** Central increased autofluorescence. **D)** Bulls eye.

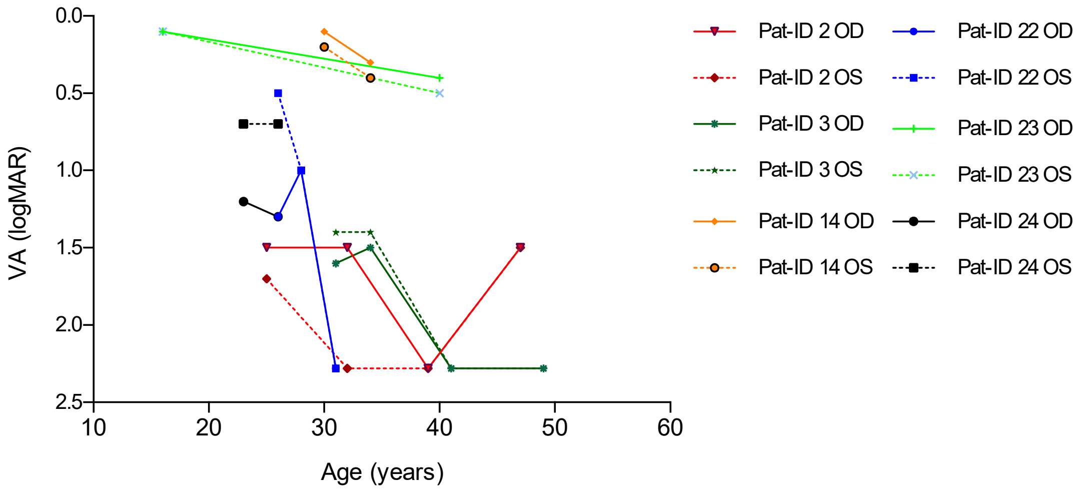

Supplemental Figure S3: Visual acuity values (OD and OS) associated with age in *GUCY2D* related AD COD/CORD patients who have been examined more than once.

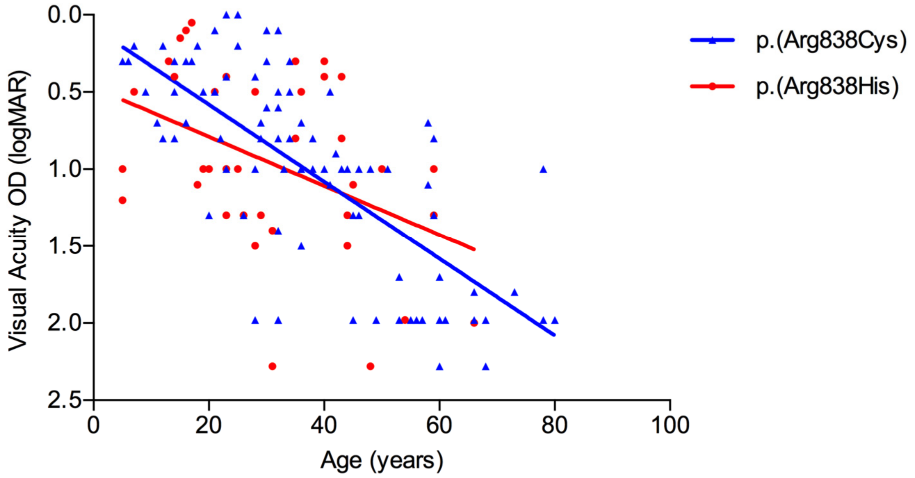

Supplemental Figure S4: Linear regression of visual acuity values (OD) associated with age in patients with p.(Arg838Cys) (n = 85) and p.(Arg838His) (n = 38).

Supplemental Table S1: Clinical data of 25 GUCY2D related AD COD/CORD patients. **(Lens 1)** 1=clear, 2=nuclear cataract, 3=cortical cataract, 4=posterior subcapsular cataract, 5=pseudophakia, 6=other; **(Fundus autofluorescence pattern 2)** 1=normal, 2=central reduced autofluorescence, 3=central increased autofluorescence, 4=bulls eye, 5=other; **(Visual field central 30° 3)** 1=normal, 2=abnormal; **(Color vision 4)** 1=normal, 2=unspecific defect, 3=tritan defect. f = female, m = male, OD = Oculus dexter, OS = Oculus sinister, N/A Not available.

Family ID	Patient ID	Sex	Protein	Mutation	Age at visit	Age at first symptoms	Major symptom at onset	BCVA (logMAR)		Lens (1)		Ocular surgeries	Systemic disease	Fundus autofluorescence pattern (2)		Visual field central 30° (3)		Visual field periphery 30°-90° (3)		Color vision (4)	
								OD	OS	OD	OS			OD	OS	OD	OS	OD	OS	OD	OS
	1	f	p.(Leu831Pro)	c.2492T>C	45	40	Glare, dark adaption difficulties	0	0	1	1	---	---	2+3	2+3	N/A	N/A	N/A	N/A	N/A	N/A
F1-III-1	2	m	p.(Arg838Gly)	c.2512C>G	25	Birth	Nystagmus, vision loss	1,5	1,5	1	1	---	---	2	2	2	2	2	2	2	2
F1-III-2	3	f	p.(Arg838Gly)	c.2512C>G	42	Birth	Vision loss	1,98	1,98	1	1	OD 2x Strabism Surgery	---	N/A	N/A	2	2	2	2	2	2
	4	f	p.(Arg838Cys)	c.2512C>T	21	18	Vision loss	0,1	0,1	1	1	---	Ulcerative colitis	N/A	N/A	2	2	2	1	2	2
F2-II-1	5	f	p.(Arg838Cys)	c.2512C>T	36	16	Vision loss	1	1	1	1	---	---	2+3	2+3	N/A	N/A	N/A	N/A	N/A	N/A
F2-III-1	6	f	p.(Arg838Cys)	c.2512C>T	18	17	Vision loss	0,2	0,2	1	1	---	---	2+3	2+3	N/A	N/A	N/A	N/A	2	2
	7	f	p.(Arg838Cys)	c.2512C>T	60	53	Vision loss	1,7	2	4	4	---	---	2+3	2+3	2	2	2	1	N/A	N/A
	8	f	p.(Arg838Cys)	c.2512C>T	30	Childhood	Vision loss	0,6	0,3	6	6	OU Lasik	---	2+3	2+3	2	2	1	1	N/A	N/A
	9	m	p.(Arg838Cys)	c.2512C>T	26	3	Vision loss	1,3	1	1	1	OU Artisan lens	---	2+3	2+3	2	2	1	1	2	2
F3-III-1	10	f	p.(Arg838Cys)	c.2512C>T	45	25	Vision loss	1,98	1,3	1	1	Strabismus surgery	---	2+3	2+3	2	2	2	2	N/A	N/A
F3-II-1	11	f	p.(Arg838Cys)	c.2512C>T	68	Childhood	Vision loss	2,28	1,98	2	2	---	Hypertension	2	2	N/A	N/A	2	2	N/A	N/A
F3-IV-1	12	m	p.(Arg838Cys)	c.2512C>T	12	12	Vision loss	0,2	0,2	1	1	---	---	2+3	2+3	2	2	1	1	N/A	N/A
	13	f	p.(Arg838Cys)	c.2512C>T	45	19	Vision loss	1,3	1,3	1	1	---	---	2	2	2	2	2	2	2	2
F4-III-1	14	m	p.(Arg838Cys)	c.2512C>T	34	N/A	N/A	0,3	0,4	1	1	OU Strabism surgery	---	3	3	2	2	2	2	2	2
F4 -II-1	15	f	p.(Arg838Cys)	c.2512C>T	58	54	Color vision, photophobia	1,1	1,1	4	2	---	Hypertension	4	4	2	2	2	2	2	2
	16	m	p.(Arg838Cys)	c.2512C>T	41	Birth	Vision loss	1,1	0,8	1	1	---	---	2	2	2	2	1	1	N/A	N/A
F5-IV-3	17	f	p.(Arg838His)*	c.2513G>A*	43	15	Vision loss	0,8	0,8	1	1	---	---	N/A	N/A	N/A	N/A	N/A	N/A	2	2
F5-V-1	18	m	p.Arg838His	c.2513G>A	17	No symptoms	No symptoms	0,05	0	1	1	---	---	N/A	N/A	N/A	N/A	N/A	N/A	N/A	N/A
F5-V-2	19	m	p.Arg838His	c.2513G>A	15	10	Vision loss	0,15	0,15	1	1	---	---	N/A	N/A	N/A	N/A	N/A	N/A	3	N/A
F5-IV-1	20	f	p.Arg838His	c.2513G>A	35	26	Vision loss	0,3	0,1	1	1	---	---	5	5	2	2	2	2	2	2
F5-III-1	21	f	p.Arg838His*	c.2513G>A*	66	30	Vision loss, color vision	2	0,7	1	2 and 4	---	Rheumatoid arthritis	2	2	N/A	N/A	N/A	N/A	5	5
	22	m	p.Arg838His	c.2513G>A	31	N/A	N/A	2,28	2,28	1	1	---	---	2	2	2	2	2	2	N/A	N/A
	23	f	p.Arg838His	c.2513G>A	40	N/A	N/A	0,4	0,5	1	1	Strabism surgery	---	4	2	2	2	1	1	1	1
	24	f	p.Arg838His	c.2513G>A	26	N/A	N/A	1,3	0,7	1	1	---	Hashimoto Thyreoiditis	1	1	2	2	1	1	2	2
	25	f	p.(Ser25Phe), p.(Thr839Arg)	c.74C>T, c.2516C>G	67	47	Glare	0,3	0,3	4	4	---	---	2+3	2+3	2	2	2	1	N/A	N/A

* In this patients an additional mutation was found (GUCA1B; p.R63X; c.187C>T). The same mutation was also found in unaffected family members and not in all affected family members.
